# Assessment of Long-Term Knowledge Retention in Children with Type 1 Diabetes and Their Families: A Pilot Study

**DOI:** 10.3390/children12081016

**Published:** 2025-08-01

**Authors:** Lior Carmon, Eli Hershkovitz, David Shaki, Tzila Gratzya Chechik, Inna Uritzki, Itamar Gothelf, Dganit Walker, Neta Loewenthal, Majd Nassar, Alon Haim

**Affiliations:** 1 Pediatrics Endocrinology Unit, Soroka University Medical Center, Beer-Sheva 8410501, Israel; elih@clalit.org.il (E.H.); davidsha@clalit.org.il (D.S.); tzilach@clalit.org.il (T.G.C.); innasha@clalit.org.il (I.U.); dganital1@clalit.org.il (D.W.); netali@clalit.org.il (N.L.); majdna@clalit.org.il (M.N.); alonhaim@clalit.org.il (A.H.); 2 Faculty of Health Sciences, Ben-Gurion University of the Negev, Beer-Sheva 8410501, Israel; gothelf@post.bgu.ac.il

**Keywords:** type 1 diabetes mellitus, education, pediatrics

## Abstract

Background: The education process for newly diagnosed Type 1 diabetes mellitus (T1D) patients and their families, primarily led by diabetes specialist nurses, is essential for gaining knowledge about the disease and its management. However, few assessment tools have been employed to evaluate long-term knowledge retention among T1D patients years after diagnosis. Methods: We developed a 20-question test to assess the knowledge of patients and their families at the conclusion of the initial education process and again 6–12 months later. Demographic and clinical data were also collected. Statistical analyses included comparisons between the first and second test results, as well as evaluation of potential contributing factors. The internal consistency and construct validity of the questionnaire were evaluated. Results: Forty-four patients completed both assessments, with a median interval of 11.5 months between them. The average score on the first test was 88.6, which declined to 82.7 on the second assessment (*p* < 0.001). In univariate analysis, factors positively associated with higher scores included Jewish ethnicity, lower HbA1c levels, and shorter hospitalization duration. Multivariate analysis revealed that parents had lower odds of experiencing a significant score decline compared to patients. Cronbach’s alpha was 0.69, and Principal Component Analysis (PCA) identified eight components accounting for 67.1% of the total variance. Conclusions: Healthcare providers should consider offering re-education to patients and their families approximately one year after diagnosis, with particular attention to high-risk populations during the initial education phase. Further studies are needed to examine this tool’s performance in larger cohorts.

## 1. Introduction

Type 1 diabetes mellitus (T1D) is an autoimmune disease with a rising global incidence over recent decades [1]. The current incidence rate among young adults (ages 10–24) is 11.07 cases per 100,000 people [2]. The disease is most commonly diagnosed during the first two decades of life, with peak onset occurring between the ages of 5–9 and 10–14 years [1].

The time of diagnosis can be especially challenging for patients—often children or adolescents—and their families. It is frequently accompanied by feelings of stress and anxiety [3,4]. Immediately following diagnosis, patients and their families are introduced to the disease and its treatment through an educational process led by a multidisciplinary team, often coordinated by a diabetes specialist nurse. Although the specifics of this education—such as setting, duration, and methodology—vary across institutions, it typically takes place in the days following diagnosis to help families return to their normal routines as soon as possible. One established method is the Diabetes Teaching and Treatment Program (DTTP), which offers five days of intensive inpatient diabetes education [5]. Since then, a variety of alternative educational strategies have been developed and assessed, including outpatient DTTP [6], the self-management–oriented education program (PRIMAS) [7], structured vignettes [8], web-based tools [9], and human patient simulation [8,10].

Previous research has highlighted the complexity of learning under stress. While some studies suggest that stressful events can enhance memory [11,12], others report memory impairments under stress or depression [12,13]. Although knowledge retention in T1D patients has been evaluated in the years following diagnosis [14,15], there is limited research assessing knowledge immediately after diagnosis. Such evaluations could provide valuable insights into the effectiveness of early education. Moreover, little is known about how knowledge changes over time in the same individuals. While caregivers often gain practical experience in disease management over time, they may also forget some of the information provided during the initial education phase.

The aim of this study was to validate a newly developed assessment tool and to evaluate the effectiveness of our educational program for newly diagnosed T1D patients and their families by comparing their knowledge immediately after the initial hospital-based training and again 6–12 months later.

## 2. Subjects and Methods

### 2.1. Participants

This was a pilot study for questionnaire validation and knowledge retention assessment. We enrolled all patients aged 0–18 years who were newly diagnosed with T1D and hospitalized at Soroka University Medical Center (SUMC) over a one-year period, from October 2022 to September 2023. Patients were excluded if they could not read Hebrew or Arabic, were transferred to another medical center immediately after diagnosis, or had one or more siblings with T1D. the sample size was derived from the number of new T1D Patients during that year.

Following diagnosis, patients are hospitalized and families attend a structured “diabetes school” coordinated by diabetes specialist nurses and involving close contact with the primary physician. Families receive instruction from diabetes nurses on key topics such as the causes and treatment of hypoglycemia, hyperglycemia, and DKA, insulin handling and administration, sick day management, and physical activity. They also meet with a dietitian to learn about nutrition and carbohydrate counting, and with a social worker. This process spans several days, and discharge occurs when both the medical team and the family feel adequately prepared. The patients in our clinic are treated either with multiple daily injections or insulin pumps.

**Figure 1:**
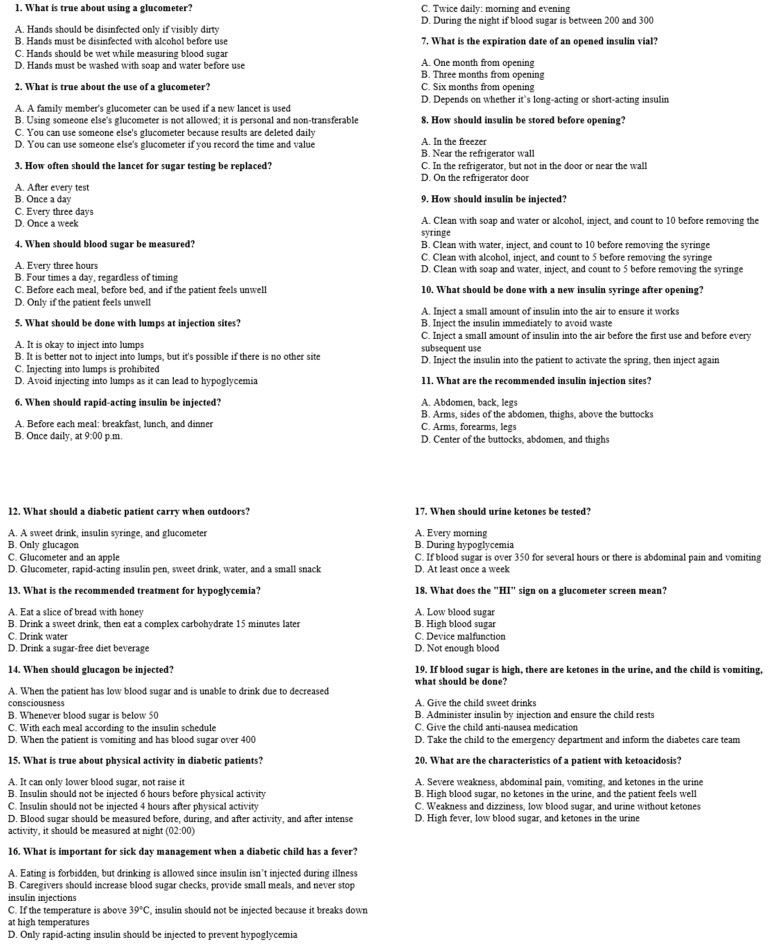
T1D knowledge questionnaire.

To assess participants’ knowledge, we developed a 20-question multiple-choice questionnaire covering key topics, including disease fundamentals; causes and treatment of hypoglycemia, hyperglycemia, and diabetic ketoacidosis (DKA); insulin storage and administration; sick-day management; and exercise guidelines (Figure 1). Each question was equally weighted at 5 points. The questionnaire was translated into Arabic, and translation accuracy was verified by back-translation into Hebrew by a different native Arabic speaker. Participants aged 13–18 years completed the questionnaire themselves, while for those aged 0–12 years, the primary caregivers completed it. The questionnaire was administered twice: first before the patient’s discharge, and again 6–12 months later during a follow-up visit at the SUMC outpatient clinic, with the same respondent completing both rounds.

Additional demographic and clinical data were extracted from electronic health records, including gender, age, ethnicity, HbA1c levels at presentation, family history of T1D (excluding siblings), and the presence of DKA at diagnosis. Informed consent was obtained from all participants who completed the questionnaire and took part in the study.

During the enrollment year, 72 new patients were diagnosed with T1D at our medical center. Of these, 53 met the inclusion criteria and completed the first questionnaire, and 44 of them also completed the second questionnaire. The remaining nine patients were lost to follow-up. The median interval between the two questionnaire administrations was 11.5 months. Out of 44 patients in the cohort, 22 (50%) were Jews and 22 (50%) were Bedouins. Other demographic and clinical characteristics of the participants are summarized in Table 1.

### 2.2. Questionnaire Validation and Statistical Analysis

To validate the questionnaire, several steps were undertaken. The internal consistency of the questionnaire was evaluated using Cronbach’s alpha, with values above 0.7 considered to indicate high reliability. To examine construct validity, a Principal Component Analysis (PCA) was conducted. PCA is a dimensionality-reduction technique used to identify clusters of related items based on inter-item correlations. Prior to PCA, the Kaiser-Meyer-Olkin (KMO) measure and Bartlett’s Test of Sphericity were used to assess sampling adequacy and the suitability of the data for factor analysis. A KMO value above 0.6 and a significant Bartlett’s test (*p* < 0.05) were considered indicators of factorability. Components with eigenvalues greater than 1 were used to determine the number of underlying factors. Test–retest reliability was assessed using the Intraclass Correlation Coefficient (ICC), calculated via a two-way mixed-effects model with absolute agreement. ICC values above 0.75 were considered to reflect good reliability, while values between 0.5 and 0.75 indicated moderate reliability.

Descriptive statistics were used to summarize patient characteristics. Categorical variables were expressed as frequencies and percentages. Normally distributed continuous variables were presented as means with standard deviations, while non-normally distributed variables were reported as medians with interquartile ranges (IQR). Sociodemographic and diabetes-related characteristics were analyzed separately for their association with scores on the first and second questionnaires. For categorical variables, subgroup mean scores were compared using either the independent *t*-test or the Mann–Whitney test, as appropriate. Continuous variables were assessed using Pearson or Spearman correlation tests. Logistic regression analysis was conducted to evaluate the hypothesis that specific sociodemographic and clinical factors were associated with score changes between the two tests. Regression coefficients and 95% confidence intervals (CIs) were calculated. Statistical analysis was performed using SPSS software (IBM, New York, NY, USA) (version 29), with a two-sided *p*-value of less than 0.05 considered statistically significant.

## 3. Results

The internal consistency of the questionnaire, measured by Cronbach’s alpha, was 0.69. To assess construct validity, a PCA with varimax rotation was performed. The KMO measure of sampling adequacy was 0.571, and Bartlett’s Test of Sphericity was significant (χ^2^ = 373.28, df = 190, *p* < 0.001), indicating that the dataset was appropriate for factor analysis. The PCA identified eight components with eigenvalues greater than one, accounting for 67.1% of the total variance. However, visual inspection of the scree plot suggested a more parsimonious three-component solution, explaining 28.7% of the variance. The intraclass correlation coefficient (ICC) for average measures was 0.695, indicating a satisfactory level of test–retest reliability and suggesting that the questionnaire provided consistent results over time. Overall, the questionnaire’s performance was acceptable, supporting its use in larger-scale studies in the future.

The mean score on the first questionnaire was 88.6, with participants answering an average of 17.6 out of 20 questions correctly, while the mean score on the second questionnaire was 82.7. This difference was statistically significant (*p* < 0.001). In univariate analysis, factors positively associated with a higher score on the first questionnaire included Jewish ethnicity, lower HbA1c levels at diagnosis, and a shorter duration of hospitalization (the latter showing a statistical trend). A higher score on the second questionnaire was also associated with shorter hospitalization duration (Table 2). In multivariate analysis, parents had lower odds of experiencing a pronounced score decline compared to patients, as shown in Table 3.

## 4. Discussion

In this study, we found that participants scored higher on the T1D knowledge examination immediately after diagnosis and completion of the educational process. However, approximately one year later, a significant decline in knowledge was observed. The questionnaire demonstrated acceptable performance, supporting its potential use in future large-scale studies.

Previous studies proposing different T1D knowledge tests have reported a wide range of results [16,17]. In our study, the initial scores were high and we attribute this to our comprehensive education process as detailed in the methods.

Differences in knowledge levels were observed among ethnic groups (Table 2), with Bedouin patients scoring lower. This may be related to lower socioeconomic status and language barriers, as some patients do not speak Hebrew. Previous studies have shown that trained interpreters and bilingual healthcare providers improve educational outcomes for non-native speakers [18]. Although interpreters or bilingual dietitians were often present during the education process, they were not consistently available, which may have impacted comprehension. Ensuring the availability of professional translators in diabetes clinics could enhance compliance and improve outcomes [19].

The socioeconomic status of Bedouins is generally lower than that of Jews [20], and socioeconomic factors are known to affect diabetes management [21]. Poorer glycemic control among Bedouins in the Negev region has been previously reported [22], and reduced knowledge may contribute to these disparities. Healthcare providers should prioritize support for groups that may be at greater risk of misunderstanding or forgetting essential information [19].

Higher HbA1c levels at diagnosis were associated with lower scores on the initial test. Elevated HbA1c reflects prolonged hyperglycemia in the months prior to diagnosis [14,23] and has been linked to lower diabetes knowledge [14,24]. Patients with higher initial HbA1c levels may have had less awareness of symptoms, which could correlate with lower baseline understanding. Initial HbA1c has been shown to predict long-term outcomes [25], and further research is needed to determine whether lower knowledge at diagnosis contributes to this relationship.

Shorter hospital stays were associated with higher scores on both tests, suggesting that these patients grasped the educational material more quickly and thoroughly. This is consistent with previous findings showing that lower health literacy is linked to longer hospitalizations [26].

Another notable finding was the greater decline in knowledge among teenage patients, who completed the questionnaires independently. Previous research has found a positive correlation between parental knowledge and younger child age [27]. It is possible that parents of younger children are more actively involved in retaining and applying diabetes knowledge over time compared to adolescents managing their own care.

Despite expectations that knowledge might improve with experience, our findings suggest that both patients and caregivers tend to forget important information over time. Several intervention strategies have been proposed. While structured education programs have not consistently improved glycemic control [28], parent support programs [29,30], self-management education [7], diabetes camps [31], and web-based tools [9,32] have all shown promise. Tailoring the educational approach to the specific needs and perceptions of each family is essential [19,33,34]. Crisis intervention program has been shown to be effective in improving newly diagnosed patients adjustment and compliance [35].

The observed decline in knowledge may have important clinical implications, including increased risk of long-term complications [14] and higher rates of DKA [36]. Diabetes educators should consider offering refresher training to all patients and caregivers one year after diagnosis, with particular attention to high-risk groups during the initial education process [37].

### Strengths and Limitations

The main strengths of this study are the development and validation of a new assessment tool, its prospective design, and longitudinal follow-up. However, limitations include its single-center setting, a specific education model that may not generalize broadly, a small sample size limiting subgroup analysis and construct validity (PCA) calculation, lack of socioeconomic data, and a relatively high rate of loss to follow-up. Given the small sample size and the nature of this study as a pilot, the results should be interpreted with caution and a degree of modesty, acknowledging the preliminary nature of the findings

## 5. Conclusions

In conclusion, while initial knowledge scores following diagnosis were relatively high, a significant decline was observed one year later. This decline may contribute to long-term complications and DKA risk. Healthcare providers should consider implementing follow-up education for patients and their families after the first year, with targeted support for high-risk populations from the outset. Our assessment tool demonstrated acceptable performance and warrants further evaluation in larger-scale, multicenter prospective studies.

## Figures and Tables

**Table 1:** The baseline characteristics of the study population (N = 44).

Characteristics		Frequencies (%)
Gender		
	Female	25 (56.8)
	Male	19 (43.2)
Ethnicity		
	Jewish	22 (50.0)
	Bedouin	22 (50.0)
Family history of diabetes		
	Yes	6 (13.6)
	No	38 (86.4)
Diabetic ketoacidosis		
	Yes (with ICU)	9 (20.5)
	Yes (without ICU)	9 (20.5)
	No	26 (59.1)
Examinee		
	Patient	13 (29.5)
	Parent	31 (70.5)
**Characteristics**		**Mean ± SD**
HbA1C at diagnosis		12.15 ± 2.71
Age of diagnosis (years)		9.41 ± 5.22
		Median ± Interquartile range
Duration of hospitalization		6.0 ± 5.0–7.0

**Table 2:** Univariate analysis of patients’ sociodemographic and clinical characteristics and the association between their average grades on the first and second tests.

Variable	Average Grade of First Test (N = 44)	*p*-Value	Average Grade of Second Test (N = 44)	*p*-Value
Total	88.64 ± 9.62		82.73 ± 13.96	<0.001
Gender		0.900		0.969
Female	88.80 ± 9.27		82.80 ± 13.39	
Male	88.42 ± 10.55		82.63 ± 15.40	
Ethnicity		0.020		0.004
Jewish	92.05 ± 5.91		88.64 ± 10.02	
Bedouin	85.23 ± 11.60		76.82 ± 15.32	
Family history of diabetes		0.599		0.846
Yes	86.67 ± 13.66		81.67 ± 13.29	
No	88.95 ± 9.16		82.89 ± 14.41	
Diabetic ketoacidosis		0.544		0.900
Yes	89.72 ± 7.37		83.06 ± 12.61	
No	87.88 ± 11.15		82.50 ± 15.31	
Examinee		0.688		0.638
Patient	90.0 ± 11.90		81.15 ± 18.27	
Parent	88.06 ± 8.82		83.39 ± 12.27	
**Variable**	**Grade of first test (correlation coefficient)**	***p*-value**	**Grade of second test (correlation coefficient)**	***p*-value**
Age of diagnosis	−0.074	0.634	−0.026	0.868
HbA1C at diagnosis	−0.308	0.045	−0.255	0.099
Duration of hospitalization	−0.256	0.093	−0.322	0.033

**Table 3:** Multivariable logistic regression analysis of sociodemographic and clinical characteristics and their association with grade difference between the two tests.

Variable	Odds Ratio for Greater Performance on the First Test; CI 95%	*p*-Value
Gender		
Female (reference)	1	
Male	0.93; 0.20–4.31	0.924
Ethnicity		
Jewish (reference)	1	
Bedouin	5.06; 0.68–37.42	0.112
Family history of diabetes		
No (reference)	1	
Yes	0.44; 0.05–3.77	0.451
Diabetic ketoacidosis		
No (reference)	1	
Yes	1.35; 0.23–8.10	0.743
Instruction recipient		
Patient (reference)	1	
Parent	0.07; 0.01–0.99	0.049
Age of diagnosis	0.83; 0.66–1.03	0.096
HbA1C at diagnosis	1.09; 0.77–1.52	0.638
Duration of hospitalization	0.96; 0.59–1.57	0.870

## Data Availability

The data presented in this study are available on request from the corresponding author due to privacy reasons.
